# Supplementary material for: YAP promotes cell-autonomous immune responses to tackle intracellular Staphylococcus aureus in vitro
Source: Nat Commun. 2022 Nov 16;13:6995. doi: 10.1038/s41467-022-34432-0 (PMC9669043; doi:10.1038/s41467-022-34432-0)
Supplement: Supplementary file 1 — Supplementary Information [file 41467_2022_34432_MOESM1_ESM.pdf]

YAP promotes cell-autonomous immune responses to tackle intracellular *Staphylococcus aureus in vitro*

Robin Caire[1,2*], Estelle Audoux[1,2], Mireille Thomas[3], Elisa Dalix[3], Aurélien Peyron[1,2], Killian Rodriguez[1,2], Nicola Pordone[1,2], Johann Guillemot[1,2], Yann Dickerscheit[1,2], Hubert Marotte[3], François Vandenesch[4,5], Frédéric Laurent[4,5], Jérôme Josse[4], Paul O. Verhoeven[1,2,6*]

[1] CIRI, Centre International de Recherche en Infectiologie, GIMAP team, Université de Lyon, Inserm, U1111, CNRS, UMR5308, ENS Lyon, Université Claude Bernard Lyon 1, Lyon, France.

[2] Faculty of Medicine, Université Jean Monnet St-Etienne, St-Etienne, France.

[3] SAINBIOSE, U1059-INSERM, Université de Lyon, St-Etienne, France

[4] CIRI, Centre International de Recherche en Infectiologie, StaPath team, Université de Lyon, Inserm, U1111, CNRS, UMR5308, ENS Lyon, Université Claude Bernard Lyon 1, Lyon, France.

[5] Department of Bacteriology, Institute for infectious Agents, Hospices Civiles de Lyon, Lyon, France.

[6] Department of Infectious Agents and Hygiene, University Hospital of St-Etienne, St-Etienne, France.

* Correspondence: Prof Paul Verhoeven. Email: paul.verhoeven@univ-st-etienne.fr; Address: Service des Agents Infectieux et d'Hygiène, CHU de St-Etienne, 42270 St Priest-en-Jarez, France. Phone/Fax number: +33 477829228/+33 477828460. Robin Caire. Email: caire.robin@gmail.com.

Supplementary Information

Supplementary Figures 1-8

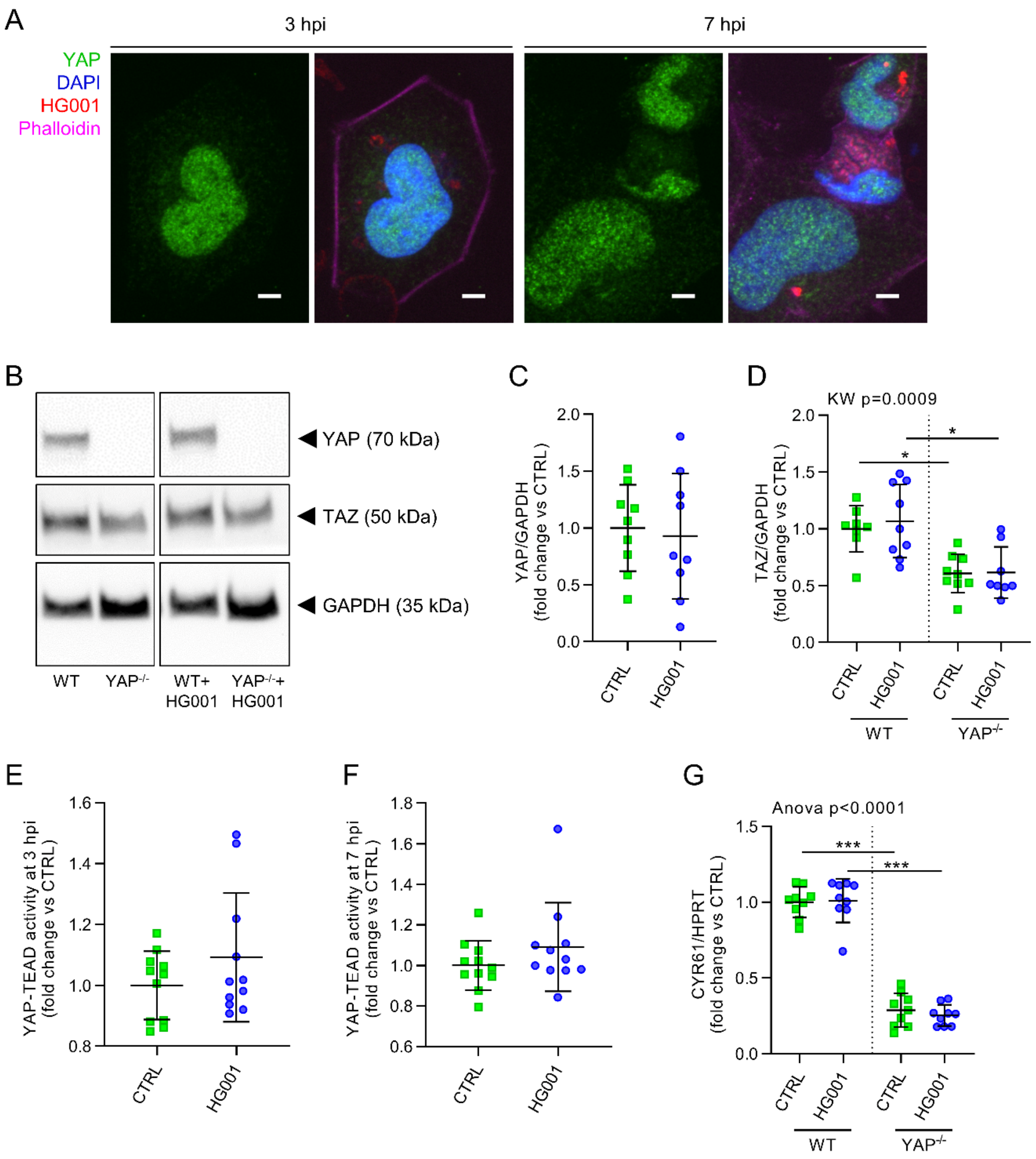

S. Figure 1. *S. aureus* HG001 strain do not inhibit YAP activity if YAP is already activated

HEK293 cells were cultured at low density (A) or medium density (B-G). Cells were infected with *S. aureus* HG001 strain at a multiplicity of infection of 10 for 7 h (or 3 h if indicated). *S. aureus* were allowed to contact for 2 h with the cells, and lysostaphin was added at 10 µg/ml for the rest of the experiments to avoid extracellular *S. aureus* multiplication. A: Confocal z-stack max intensity projection images of YAP (immunolabeling, green), DAPI (nuclei, blue), DsRed-expressing *S. aureus* (red), and phalloidin (actin filament, magenta); images are representative of three independent experiments; scale bar: 5 µm. B-D: Representative immunoblot results of YAP, TAZ, and GAPDH (B) with their quantification normalized by GAPDH expression (C and D) (C: n=9/group; D: WT CTRL n=8, $YAP^{-/-}$ CTRL n=9, WT HG001 n=9, $YAP^{-/-}$ HG001 n=8). E-F: Luciferase reporter assay of TEAD transcription factor activity (8xGTIIC) for HG001 *S. aureus* infection at 3 hpi (E) or 7 hpi (F) (n=11/group). G: RT-qPCR quantification of CYR61 expression normalized to HPRT expression (n=9/group). Results are expressed as fold change vs. control group and presented as individual values with mean ± SD, and representative of three independent experiments. One-way analysis of variance (ANOVA) or Kruskal-Wallis (KW) test with FDR correction for multiple comparisons post hoc tests: * $p < 0.05$; *** $p < 0.001$.

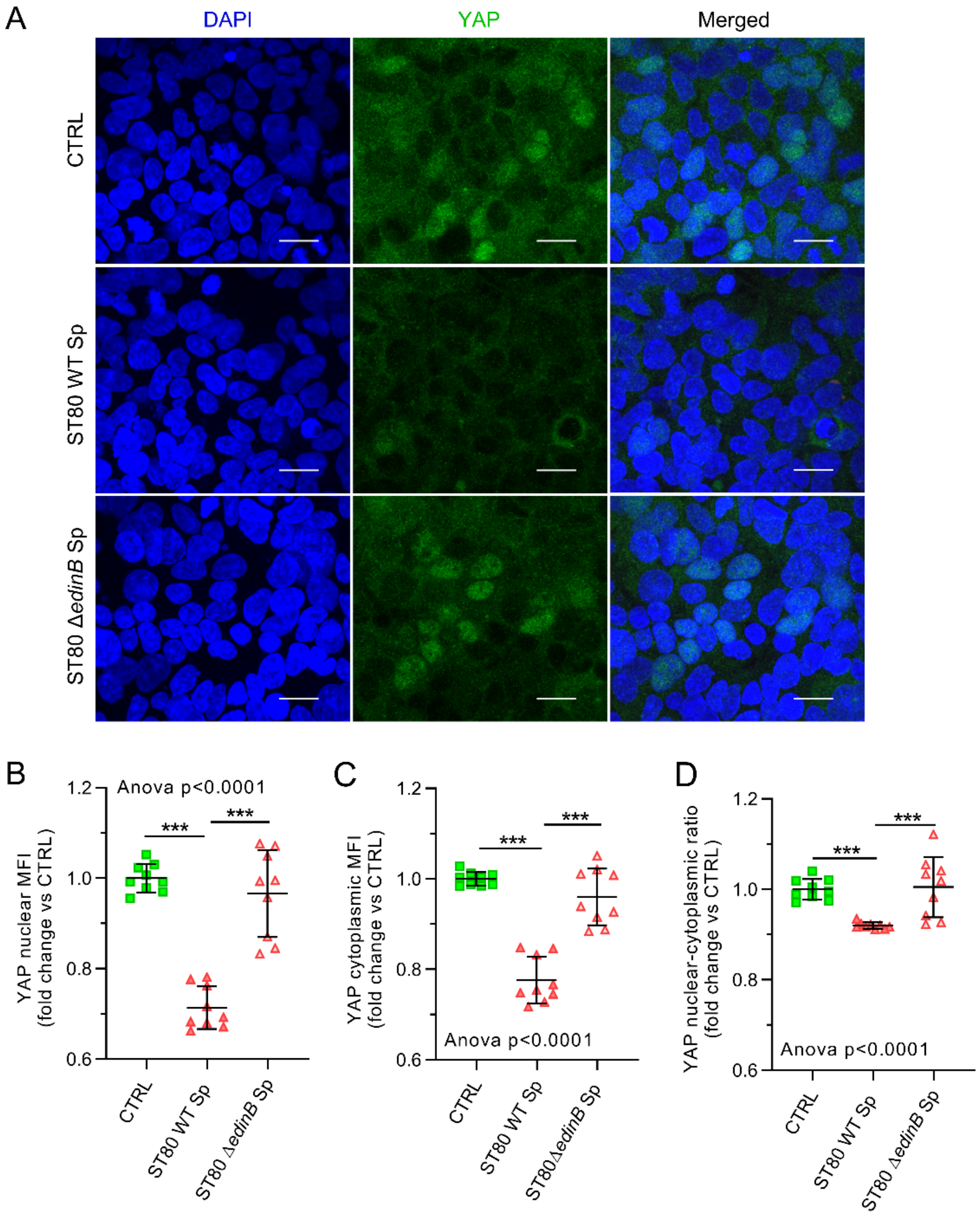

S. Figure 2. *S. aureus* supernatant containing EDIN-B prevented YAP activation in HEK293 cells
HEK293 cells cultured at high density were treated with supernatant of *S. aureus* ST80 WT and ST80 ΔedinB for 24 h. A: Confocal representative z-stack max intensity projection images of cells labelled with anti-YAP antibody (green), DAPI (nuclei, blue). Scale bar: 20 µm. B-D: Quantification of YAP nuclear mean fluorescence intensity (MFI) (B), YAP cytoplasmic MFI (C), and YAP nuclear cytoplasmic ratio (D) (n=9/group). Results are expressed as fold change vs. control group and presented as individual values with mean ± SD, representing three independent experiments. CTRL: control; WT: wild-type; ST80Δ*edinB*: EDIN-B-deleted ST80 strain; Sp: supernatant. One-way analysis of variance (ANOVA) test with FDR correction for multiple comparison post-hoc tests: *** $p < 0.001$.

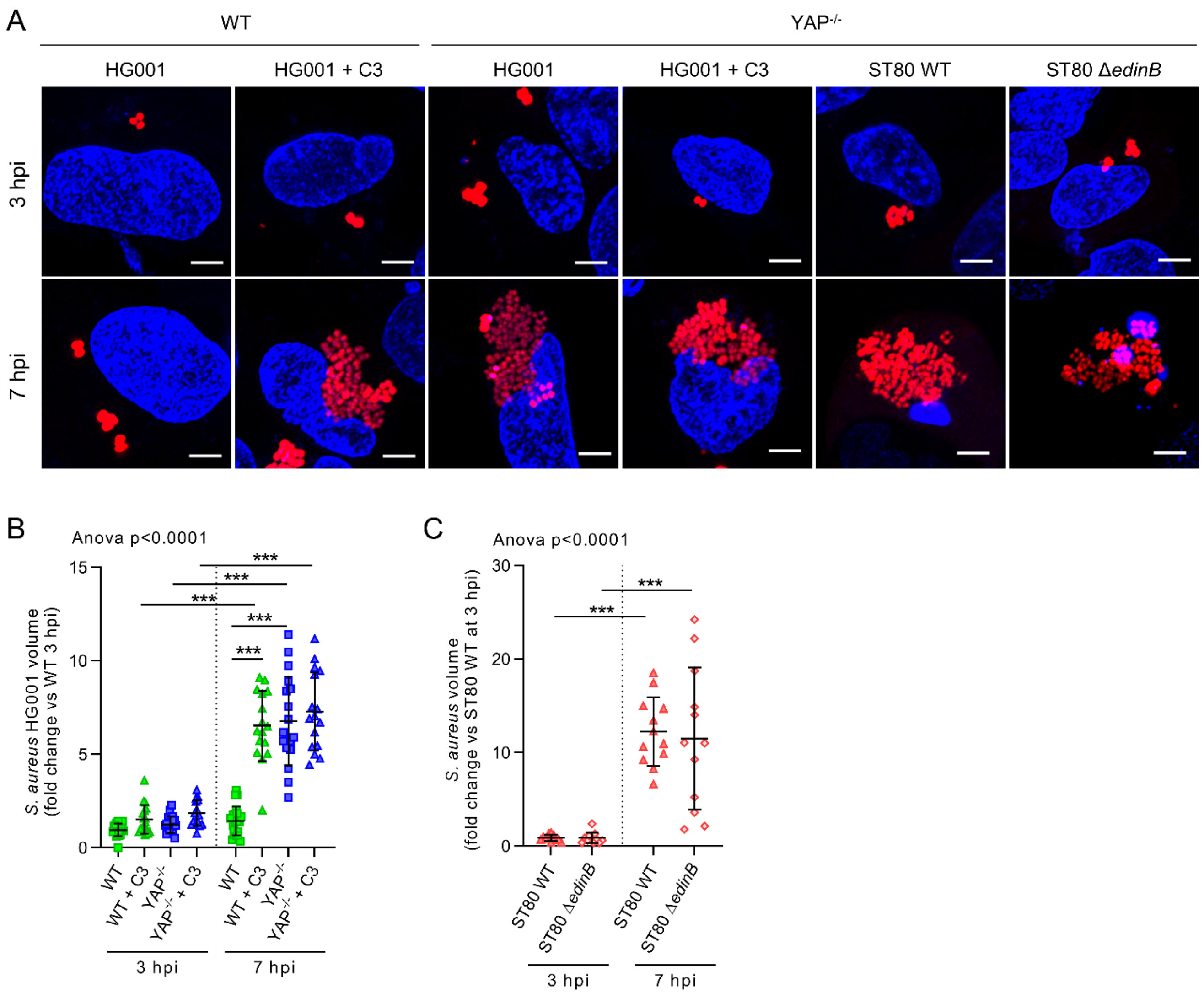

S. Figure 3. RhoA-inhibiting toxins promote *S. aureus* intracellular growth through YAP inhibition

HEK293 cells were cultured at medium density and infected with *S. aureus* HG001 strain at a multiplicity of infection of 1 for 3 or 7 h, as indicated. *S. aureus* were allowed to contact cells for 2 h, and lysostaphin was added at 10 µg/mL for the remaining experiments to avoid extracellular *S. aureus* multiplication. Cell permeable C3 exoenzyme (1 µg/mL) from *C. botulinum* was added to the cells with *S. aureus* HG001 during the initial 2 h and during lysostaphin treatment. A: Representative confocal 10 z-stack maximum intensity projection images of live cells labeled with Hoechst (nuclei, blue) and infected with DsRed-expressing *S. aureus* HG001 or ST80 strains (red); scale bar: 5 µm. B, C: Corresponding quantification of intracellular HG001(B) and ST80 (C) mean volume per cell (B: WT 3 hpi n=20, WT + C3 3 hpi n=18, YAP$^{-/-}$ 3 hpi n=21, YAP$^{-/-}$ + C3 3 hpi n=16, WT 7 hpi n=22, WT + C3 7 hpi n=16, YAP$^{-/-}$ 7 hpi n=18, YAP$^{-/-}$ + C3 7 hpi n=16; C: n=9/group). Results are expressed as fold change vs. control group and presented as individual values with mean ± SD, representing three independent experiments. WT: wild-type. One-way analysis of variance (ANOVA) test with FDR correction for multiple comparison post-hoc tests: * $p < 0.05$, ** $p < 0.01$, *** $p < 0.001$.

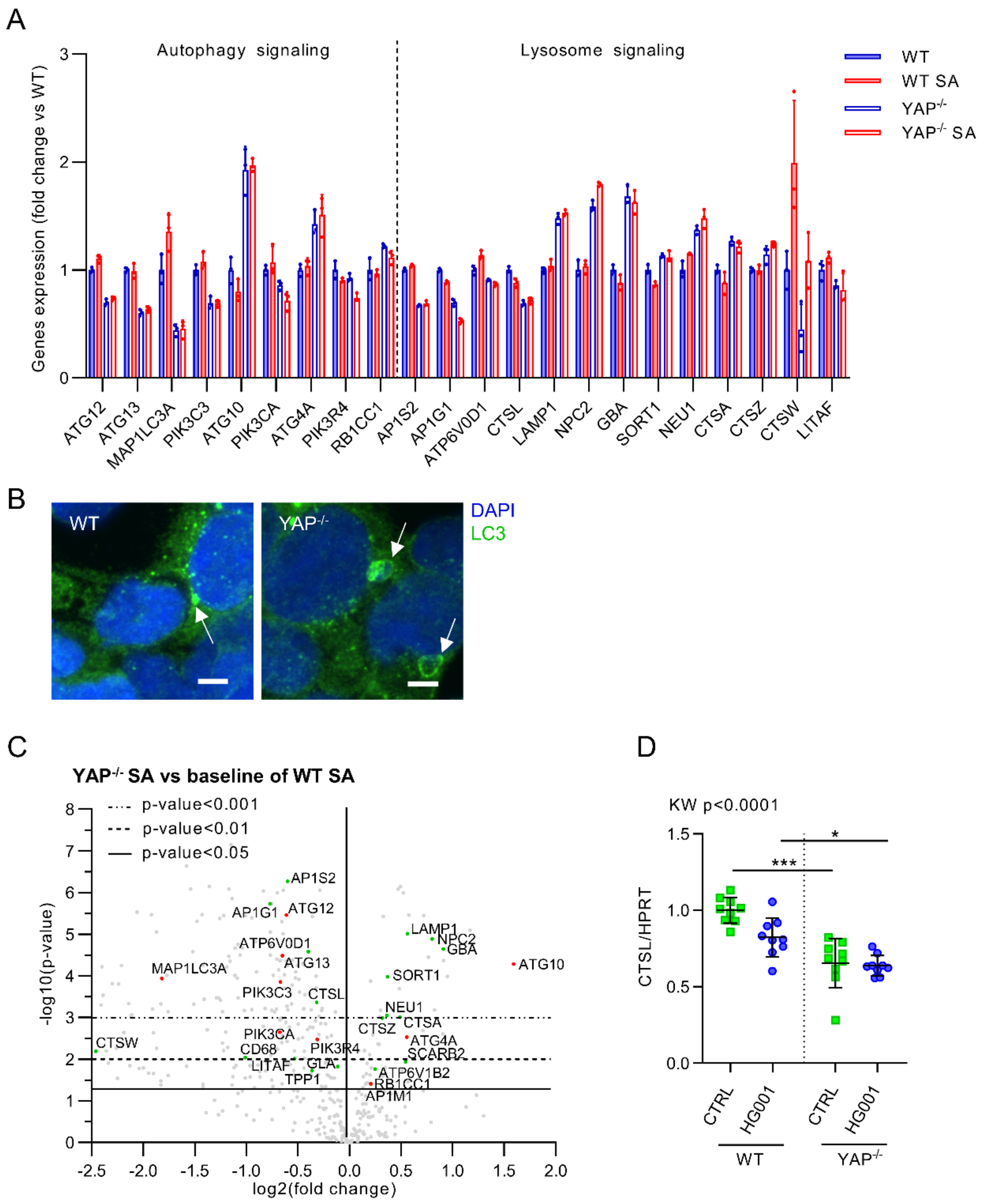

S. Figure 4. Autophagy lysosome pathways regulation during infection in WT and YAP$^{-/-}$ cells

HEK293 cells were cultured at medium density and infected with *S. aureus* HG001 strain at a multiplicity of infection of 10 for 7 h. *S. aureus* were allowed to contact cells for 2 h with the cells, then, lysostaphin was added at 10 µg/ml for the remaining experiment to avoid extracellular *S. aureus* multiplication. A: nCounter Nanostring host response autophagy and lysosome gene expression in the four groups; depicted genes were selected if at least one comparison between two groups gave a corrected p-value < 0.01 (n=3/group). B: Confocal z-stack max intensity projection images of LC3 immunolabeling (autophagic vesicles, green) and DAPI (nuclei, blue); images are representative of three independent experiments; scale bar: 5 µm. C: Volcano plot representation of differential gene expression in YAP$^{-/-}$ infected group versus the baseline of WT infected group; depicted genes are lysosome (green) and autophagy (red) related pathway genes differentially expressed. D: RT-qPCR quantification of CTSL expression normalized to HPRT expression (n=9/group). WT: Wild type; SA: *S. aureus*. Results are expressed as fold change vs. control group and presented as histograms (A) or individual values (D) with mean ± SD, and representative of three independent experiments (D). One-way analysis of variance (ANOVA) or Kruskal-Wallis (KW) test with FDR correction for multiple comparisons post hoc tests (D): * $p < 0.05$; *** $p < 0.001$. The p-values were calculated using nanostring advanced software based on t-test corrected with false discovery rate (C).

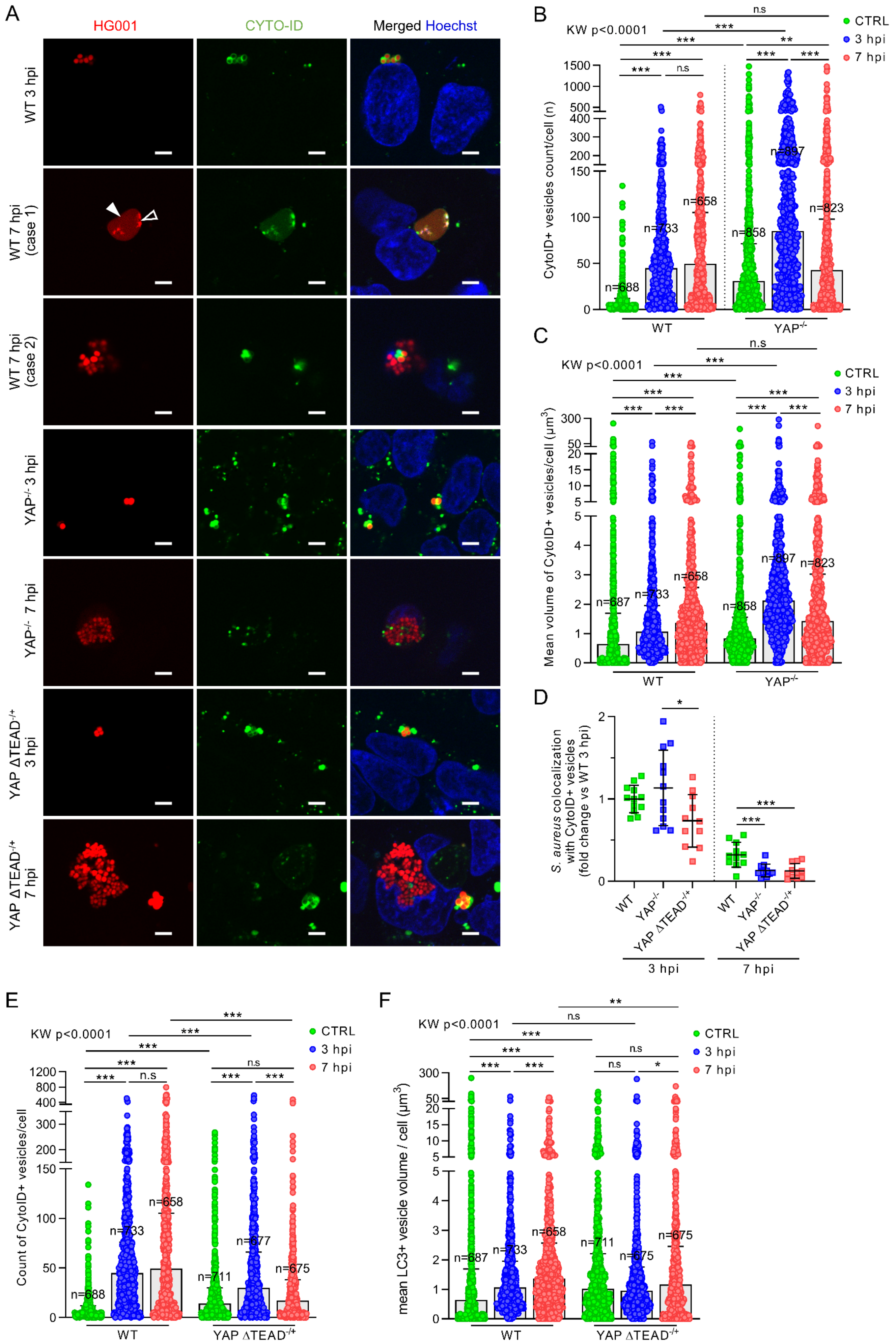
A
HG001
CYTO-ID
Merged Hoechst
WT 3 hpi
WT 7 hpi (case 1)
WT 7 hpi (case 2)
YAP-/- 3 hpi
YAP-/- 7 hpi
YAP ΔTEAD-/+ 3 hpi
YAP ΔTEAD-/+ 7 hpi
B
KW p<0.0001
CytoID+ vesicles count/cell (n)
n=688
n=733
n=658
n=858
n=897
n=823
WT
YAP-/-
CTRL
3 hpi
7 hpi
C
KW p<0.0001
Mean volume of CytoID+ vesicles/cell (μm3)
n=687
n=733
n=658
n=858
n=897
n=823
WT
YAP-/-
D
S. aureus colocalization with CytoID+ vesicles (fold change vs WT 3 hpi)
WT
YAP-/-
YAP ΔTEAD-/+
3 hpi
7 hpi
E
KW p<0.0001
Count of CytoID+ vesicles/cell
n=688
n=733
n=658
n=711
n=677
n=675
WT
YAP ΔTEAD-/+
F
KW p<0.0001
mean LC3+ vesicle volume / cell (μm3)
n=687
n=733
n=658
n=711
n=675
n=675
WT
YAP ΔTEAD-/+

S. Figure 5. YAP/TEAD transcriptional activity is required to reduce *Staphylococcus aureus* induced autophagic flux blockage

HEK293 cells cultured at medium density and were infected with *S. aureus* HG001 strain at a multiplicity of infection of 1 for 3 or 7 h, as indicated. *Staphylococcus aureus* were allowed to contact cells for 2 h, and lysostaphin was added at 10 µg/mL for the remaining experiments to avoid extracellular *S. aureus* multiplication. A: Representative confocal (0.5 µm thick z-stack) images of live cells labeled with Hoechst (nuclei, blue), CYTO-ID (autophagic vesicles, green), and DsRed-expressing *S. aureus* (red), and merged; white arrowhead: diffused red fluorescence within autophagic vesicles. White empty arrowhead: disrupted *S. aureus.* Note that WT-infected cells at 7 hpi are represented in two different cases. Scale bar: 5 µm. B-C: Corresponding quantification in WT and $YAP^{-/-}$ cells of the CYTO-ID-positive vesicle count (B) or mean volume (C) per cell as indicated. Each point represents one cell. The number of analyzed cells per group is shown. D: Quantification of the relative percentage of colocalization between *S. aureus* and CYTO-ID labelling (autophagic vesicles) (WT 3 hpi n=12, $YAP^{-/-}$ 3 hpi n=12, $YAP\Delta TEAD^{-/+}$ 3 hpi n=11, WT 7 hpi n=11, $YAP^{-/-}$ 7 hpi n=11, $YAP\Delta TEAD^{-/+}$ 7 hpi n=11). E, F: Corresponding quantification in WT and $YAP\Delta TEAD^{-/+}$ cells of CYTO-ID-II-positive vesicles (B) or mean volume (C) per cell. Each point represents one cell. The number of analyzed cells per group is shown. Results are presented as individual values with mean ± SD (D) or median with interquartile range, representing three independent experiments. WT: Wild type. One-way analysis of variance (ANOVA) or Kruskal-Wallis (KW) test with false discovery rate (FDR) correction for multiple comparisons post hoc tests: * $p < 0.05$, ** $p < 0.01$, *** $p < 0.001$.

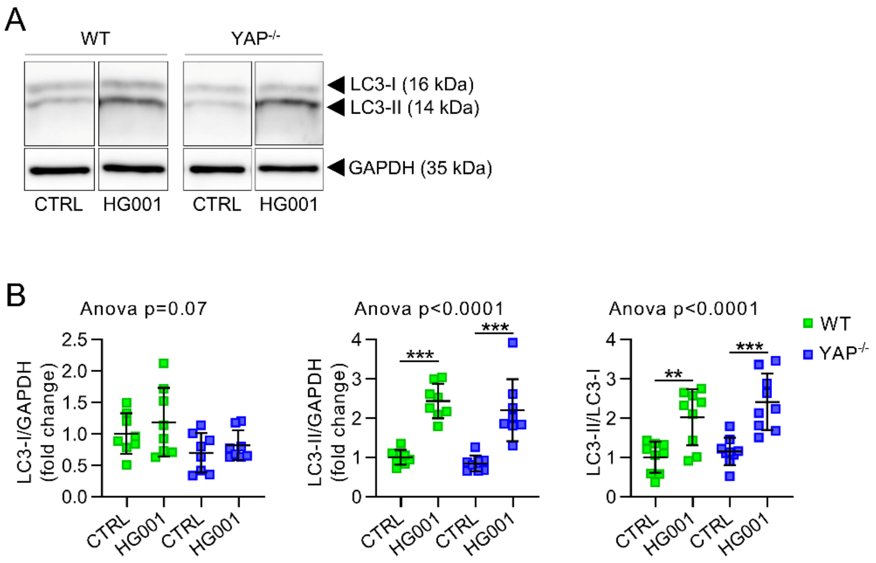

S. Figure 6. YAP/TEAD transcriptional activity is required to reduce *Staphylococcus aureus*-induced autophagic flux blockage

HEK293 cells were cultured at medium density and infected with *S. aureus* HG001 strain at a multiplicity of infection of 10 for 7 h. *S. aureus* were allowed to contact cells for 2 h, and lysostaphin was added at 10 µg/mL for the remaining experiments to avoid extracellular *S. aureus* multiplication. A, B: Representative immunoblots for LC3-I and -II, and GAPDH (A), with their quantification normalized to GAPDH expression (B) (LC3-I / GAPDH: WT CTRL n=8, WT HG001 n=8, YAP-/- CTRL n=8, YAP-/- HG001 n=8; LC3-II / GAPDH: WT CTRL n=8, WT HG001 n=8, YAP-/- CTRL n=8, YAP-/- HG001 n=8; LC3-II / LC3-I: WT CTRL n=9, WT HG001 n=9, YAP-/- CTRL n=9, YAP-/- HG001 n=9). Results are presented as individual values with mean ± SD representing three independent experiments. WT: Wild type. One-way analysis of variance (ANOVA) test with false discovery rate (FDR) correction for multiple comparisons post hoc tests: * $p < 0.05$, ** $p < 0.01$, *** $p < 0.001$.

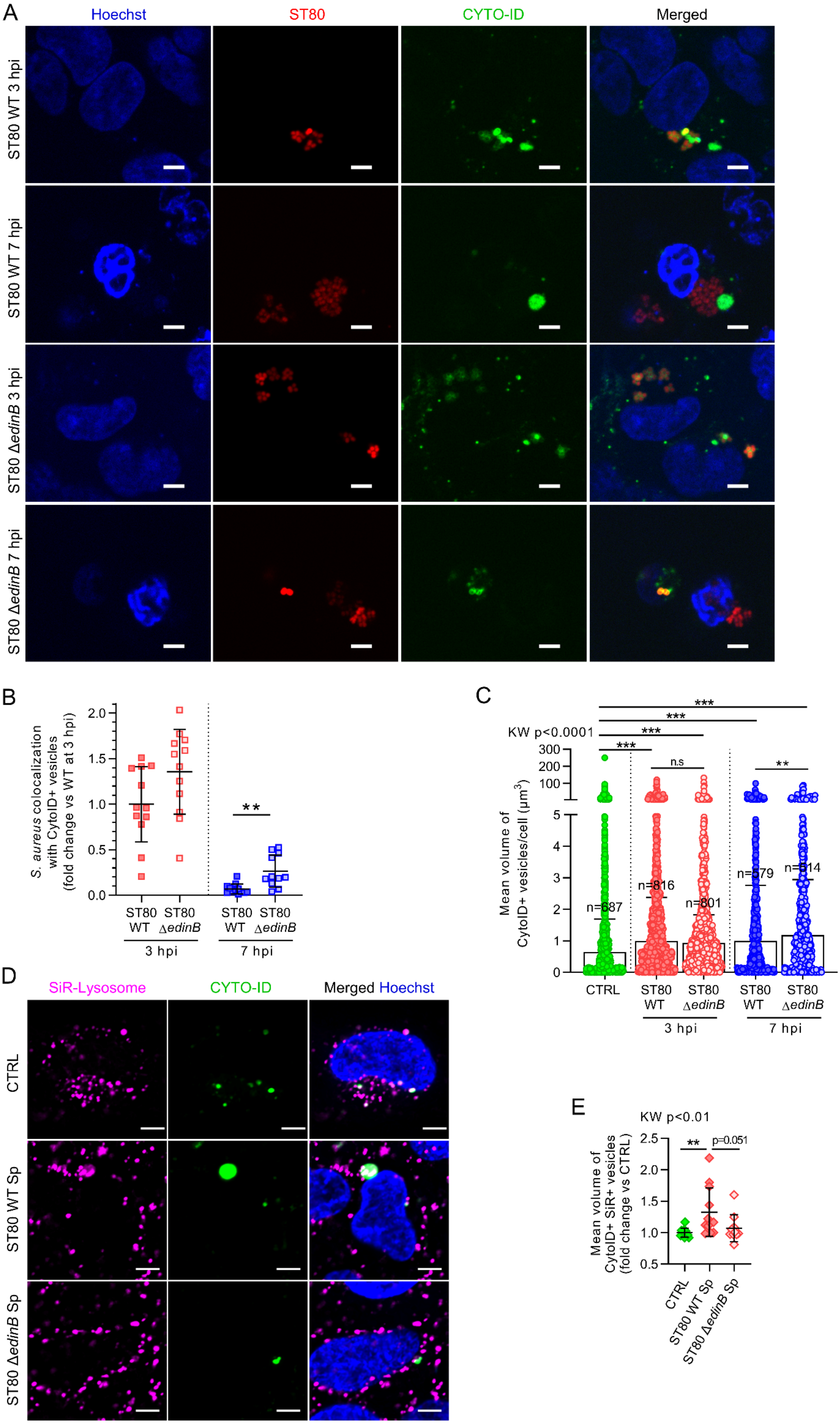

A
Hoechst
ST80
CYTO-ID
Merged
ST80 WT 3 hpi
ST80 WT 7 hpi
ST80 ΔedinB 3 hpi
ST80 ΔedinB 7 hpi
B
S. aureus colocalization with CytoID+ vesicles (fold change vs WT at 3 hpi)
2.0
1.5
1.0
0.5
0.0
**
ST80 WT
ST80 ΔedinB
3 hpi
7 hpi
C
KW p<0.0001
***
n.s
**
Mean volume of CytoID+ vesicles/cell (µm3)
300
200
100
n=687
n=816
n=801
n=579
n=614
CTRL
ST80 WT
ST80 ΔedinB
3 hpi
7 hpi
D
SiR-Lysosome
CYTO-ID
Merged Hoechst
CTRL
ST80 WT Sp
ST80 ΔedinB Sp
E
KW p<0.01
p=0.051
Mean volume of CytoID+ SiR+ vesicles (fold change vs CTRL)
2.5
2.0
1.5
1.0
0.5
CTRL
ST80 WT Sp
ST80 ΔedinB Sp

S. Figure 7. EDIN B expression by *S. aureus* promotes its escape from autophagic vesicles

HEK293 cells were cultured at medium density and infected with *S. aureus* ST80 WT and ST80 Δ*edinB* strains at a multiplicity of infection of 1 for 3 or 7 h, as indicated. *S. aureus* were allowed to contact for 2 h with the cells, and lysostaphin was added at 10 µg/ml for the rest of the experiments to avoid extracellular *S. aureus* multiplication. A: Representative confocal (0.5 µm thick z-stack) images of live cells labeled with Hoechst (nuclei, blue), CYTO-ID (autophagic vesicles, green), DsRed-expressing *S. aureus* (red); scale bar: 5 µm. B: Quantification of the relative percentage of colocalization between *S. aureus* and CYTO-ID labelling (autophagic vesicles) (ST80 WT 3 hpi n=12, ST80 Δ*edinB* 3 hpi n=12, ST80 WT 7 hpi n=12, ST80 Δ*edinB* 7 hpi n=11). The results were expressed as fold change vs. the WT 3 hpi group set at 100%. C: corresponding quantification of the mean volume of CYTO-ID-positive vesicle per cell. Each point represents one cell. The number of analyzed cells per group is shown. D: Representative confocal (0.5 µm thick z-stack) images of live cells labeled with Hoechst (nuclei, blue), CYTO-ID (autophagic vesicles, green), and SiR-Lysosome (lysosome, magenta). Cells were treated with supernatant from the ST80 strain for 24 h. E: Corresponding quantification of mean volume of CYTO-ID and SiR-Lysosome double-positive vesicles (vesicle volume/vesicle count) (CTRL n=11, ST80 WT Sp n=12, ST80 Δ*edinB* Sp n=10). Results are expressed as fold change vs. control group and presented as individual values with mean ± SD (B and E), or median with interquartile range representing three independent experiments. T-test (B) or Kruskal-Wallis (KW) test with FDR correction for multiple comparisons post hoc tests: * $p < 0.05$, ** $p < 0.01$, *** $p < 0.001$.

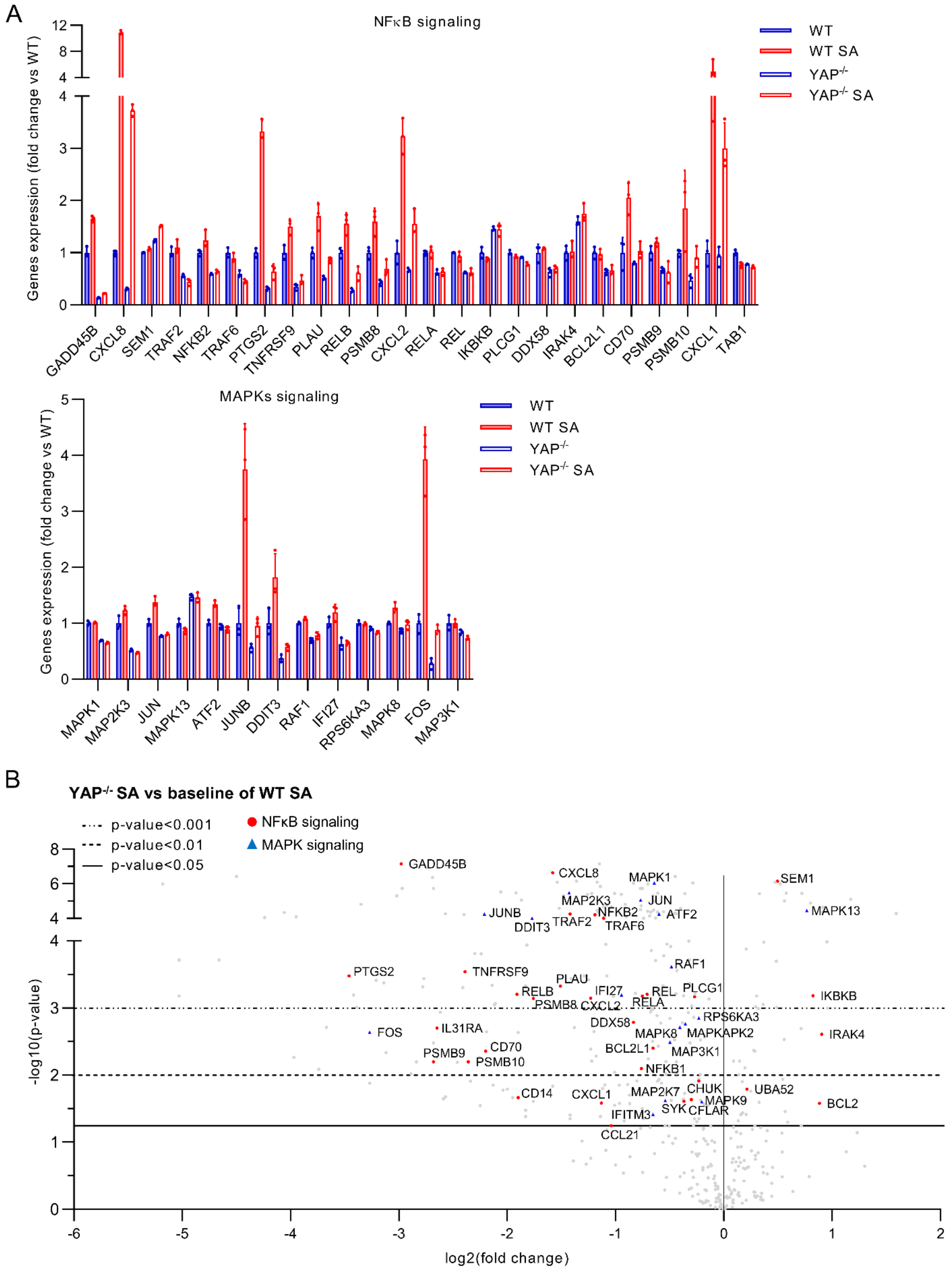

S. Figure 8. YAP promotes inflammatory response during *S. aureus* infection

HEK293 cells were cultured and infected, as described in Supplementary Figure 3. A: nCounter Nanostring host response for NF-κB and MAPK gene expression in the four groups; depicted genes were selected if at least one comparison between two groups gave a corrected p-value < 0.01. Results are expressed as histograms with mean ± SD. B: Volcano plot representation of differential gene expression in YAP$^{-/-}$ infected group versus the baseline of WT infected group; depicted genes are NF-κB (red circle) and MAPK (blue triangle) related pathway genes differentially expressed (n=3/group). The p-value was calculated using nanostring advanced software based on a t-test corrected with false discovery rate. WT: Wild type; SA: *S. aureus*.
