# Supplementary material for: YAP promotes cell-autonomous immune responses to tackle intracellular Staphylococcus aureus in vitro
Source: Nat Commun. 2022 Nov 16;13:6995. doi: 10.1038/s41467-022-34432-0 (PMC9669043; doi:10.1038/s41467-022-34432-0)
Supplement: Supplementary file 2 — Reporting Summary [file 41467_2022_34432_MOESM2_ESM.pdf]

nature portfolio

Corresponding author(s): Paul O. Verhoeven
Robin Caire

Last updated by author(s): 14/10/2022

Reporting Summary

Nature Portfolio wishes to improve the reproducibility of the work that we publish. This form provides structure for consistency and transparency in reporting. For further information on Nature Portfolio policies, see our Editorial Policies and the Editorial Policy Checklist.

Statistics

For all statistical analyses, confirm that the following items are present in the figure legend, table legend, main text, or Methods section.

n/a	Confirmed	
☐	☒	The exact sample size (*n*) for each experimental group/condition, given as a discrete number and unit of measurement
☐	☒	A statement on whether measurements were taken from distinct samples or whether the same sample was measured repeatedly
☐	☒	The statistical test(s) used AND whether they are one- or two-sided<br>*Only common tests should be described solely by name; describe more complex techniques in the Methods section.*
☐	☒	A description of all covariates tested
☐	☒	A description of any assumptions or corrections, such as tests of normality and adjustment for multiple comparisons
☐	☒	A full description of the statistical parameters including central tendency (e.g. means) or other basic estimates (e.g. regression coefficient) AND variation (e.g. standard deviation) or associated estimates of uncertainty (e.g. confidence intervals)
☐	☒	For null hypothesis testing, the test statistic (e.g. *F*, *t*, *r*) with confidence intervals, effect sizes, degrees of freedom and *P* value noted<br>*Give P values as exact values whenever suitable.*
☒	☐	For Bayesian analysis, information on the choice of priors and Markov chain Monte Carlo settings
☒	☐	For hierarchical and complex designs, identification of the appropriate level for tests and full reporting of outcomes
☒	☐	Estimates of effect sizes (e.g. Cohen's *d*, Pearson's *r*), indicating how they were calculated

Our web collection on statistics for biologists contains articles on many of the points above.

Software and code

Policy information about availability of computer code

Data collection	The following software was used: NIS software (v5.30, Nikon); Fiji software (v1.52p, NIH, USA)
Data analysis	The following softwares were used: NIS software (v5.30, Nikon); nSolver software (v4.0, NanoString Technology) and nSolver Advance Analysis Module (v2.0.134, NanoString Technology); GraphPad software (v9.3.0, Prism)

For manuscripts utilizing custom algorithms or software that are central to the research but not yet described in published literature, software must be made available to editors and reviewers. We strongly encourage code deposition in a community repository (e.g. GitHub). See the Nature Portfolio guidelines for submitting code & software for further information.

Data

Policy information about availability of data

All manuscripts must include a data availability statement. This statement should provide the following information, where applicable:
- Accession codes, unique identifiers, or web links for publicly available datasets
- A description of any restrictions on data availability
- For clinical datasets or third party data, please ensure that the statement adheres to our policy

The data generated in this study are provided in the Source Data file. The Nanostring nCounter data generated in this study have been deposited in the Gene Expression Omnibus (GEO) database under accession code GSE197181: https://www.ncbi.nlm.nih.gov/geo/query/acc.cgi?acc=GSE197181

Human research participants

Policy information about studies involving human research participants and Sex and Gender in Research.

Reporting on sex and gender	Sex: Female. Gender: not reported.
Population characteristics	Human primary cells (Fibroblast-like synoviocytes) used in this study were collected from 3 different osteoarthritic patients undergoing surgery. All were females with a median age of 62 years old.
Recruitment	The fibroblast-like synoviocytes were obtained during hip or knee replacement surgery. Inclusion Criteria: Age > 18; Osteoarthritis with joint surgery planned; Informed consent approved and signed. Exclusion Criteria: Pregnancy.
Ethics oversight	This protocol was approved by the ethical committee of the University Hospital of St-Etienne (France) and register under the following accession number: IRB # 2014-A01688-39.

Note that full information on the approval of the study protocol must also be provided in the manuscript.

Field-specific reporting

Please select the one below that is the best fit for your research. If you are not sure, read the appropriate sections before making your selection.

☒ Life sciences ☐ Behavioural & social sciences ☐ Ecological, evolutionary & environmental sciences

For a reference copy of the document with all sections, see nature.com/documents/nr-reporting-summary-flat.pdf

Life sciences study design

All studies must disclose on these points even when the disclosure is negative.

Sample size	No statistical method was used to predetermine sample size. All data were obtained from at least 3 independant experiment (except for NanoString experiment done once). For each experiments 2 to 6 biological replicates were included in experimental groups. This design is suited to take into account the noise induced by random biological variation. By pooling the 3 independent experiments, we obtained 8 to 18 samples per group, which we was enough for the statistical tests performed in this study.
Data exclusions	No data were excluded. In case of poor sample quality (low RNA quality, low number of cells/well) making the experiment impossible, the samples were not included in the independent experiments.
Replication	All data are representative of at least 3 independant experiments. We find reproducible results between each independant experiments.
Randomization	Samples were randomly assigned to groups.
Blinding	For cell culture experiments investigators were not blinded because of the inherent need to know group allocation to treat the cells accordingly. Investigators were partially blinded for data collection because all samples were assigned with numbers instead of full names. All samples were always collected identically. For microscopy experiments, investigators were partially blinded to group allocation since microscopy fields imaged were selected randomly for each groups and each groups were named as numbers instead of full names. For data analysis blinding was not relevant because all the analysis performed were based on automatic quantifications.

Reporting for specific materials, systems and methods

We require information from authors about some types of materials, experimental systems and methods used in many studies. Here, indicate whether each material, system or method listed is relevant to your study. If you are not sure if a list item applies to your research, read the appropriate section before selecting a response.

Materials & experimental systems

n/a	Involved in the study	
☐	☒	Antibodies
☐	☒	Eukaryotic cell lines
☒	☐	Palaeontology and archaeology
☒	☐	Animals and other organisms
☒	☐	Clinical data
☒	☐	Dual use research of concern

Methods

n/a	Involved in the study	
☒	☐	ChIP-seq
☒	☐	Flow cytometry
☒	☐	MRI-based neuroimaging

Antibodies

Antibodies used	YAP antibody (63.7 sc-101199, Santa Cruz Biotechnology; 1:100), rabbit anti-LC3A/B antibody (4108, Cell Signaling Technology, Leiden, The Netherlands 1:100), mouse and rabbit IgG isotype antibody (31903 and 31235, Thermo Fisher Scientific; used at the same concentration as YAP or LC3A/B antibodies); secondary antibody, goat anti-mouse 488 or goat anti-rabbit 488 (A11034 and A32731, Thermo Fisher; 1:400); For western blot, primary antibodies purchased from Cell Signaling Technology (Danvers, MA, USA) diluted at 1:1,000: YAP/TAZ (#8418), YAP (##14074), LC3A/B (#12741), and 1:5,000: GAPDH (#2118).
Validation	All antibodies used in this study were validated by manufacturers for targeting human proteins (we only use human cells) and for the application we used in this study (e.g., Western blot and/or IHC/IF). All antibodies used are well described and commonly used in the litterature. Moreover all the YAP targeting antibodies we used were specific for human YAP since HEK293 YAP deficient cells were not labelled by these antibodies (IF for 63.7 or WB for others). For IF, controls were always performed with isotypic antibodies to validate the specificity of the signal.

Eukaryotic cell lines

Policy information about cell lines and Sex and Gender in Research

Cell line source(s)	HEK293 were obtained from ATCC; Fibroblast-like synoviocytes (FLS) were collected from humans.
Authentication	No authentication procedures. HEK-293 are not known misidentified cell lines maintained by the International Cell Line Authentication Committee.
Mycoplasma contamination	All cells used in this study were routinely tested from mycoplasma and were mycoplasma free.
Commonly misidentified lines (See ICLAC register)	None.
